# Lead exposure promotes *NF2-wildtype* meningioma cell proliferation through the Merlin-Hippo signaling pathway

**DOI:** 10.1265/ehpm.24-00216

**Published:** 2025-02-01

**Authors:** Nenghua Zhang, Xiaohua Shen, Yunnong Yu, Long Xu, Zheng Wang, Jia Zhu

**Affiliations:** 1 Department of Immunology, College of Medicine, Jiaxing University, Jiaxing, 314001, China; 2 Department of Experimental Diagnosis, Provincial and Municipal Medical Key Disciplines, Jiaxing University Affiliated Traditional Chinese Medicine Hospital, Jiaxing 314000, China; 3 School of International and Public Affairs, Shanghai Jiao Tong University, Shanghai 200240, China; 4 Department of Public Health, College of Medicine, Jiaxing University, Jiaxing, 314001, China

**Keywords:** Lead exposure, Meningioma, Proliferation, Merlin, Hippo signaling pathway, YAP

## Abstract

**Background:**

Lead is a persistent inorganic environmental pollutant with global implication for human health. Among the diseases associated with lead exposure, the damage to the central nervous system has received considerable attention. It has been reported that long-term lead exposure increases the risk of meningioma; however, the underlying mechanism remains poorly understood. Clinical studies have indicated that loss-of-function and mutations in the *neurofibromin-2 (NF2)* gene play a crucial role in promoting meningioma formation.

**Methods:**

The effect of Pb on meningioma were tested *in-vitro* and *in-vivo*. Two human meningioma cell lines were used in this study, including *NF2-wildtype* IOMM-Lee cell and *NF2-null* CH157-MN cell. Cell viability, cell cycle and cell size were examined after Pb exposure. The expression of Merlin, mammalian sterile 20-like kinases 1 and 2 (MST1/2) and Yes-associated protein (YAP) from these two meningioma cells were analyzed by Western blot. A xenograft mouse model was constructed by subcutaneous injection of IOMM-Lee meningioma cells.

**Results:**

This study demonstrated that treatment with lead induce dose-dependent proliferation in IOMM-Lee cell (with an EC_50_ value of 19.6 µM). Moreover, IOMM-Lee cell exhibited augmented cell size in conjunction with elevated levels of phosphorylated histone H3, indicative of altered cell cycle progression resulting from lead exposure. However, no significant change was observed in the CH157-MN cell. Additionally, the Merlin-Hippo signaling pathway was inactivated with decreased Merlin and phosphorylation levels of MST1/2 and YAP, leading to increased YAP nuclear translocation in IOMM-Lee cells. However, there was no change in the Merlin-Hippo signaling pathway in CH157-MN cells after lead treatment. The administration of Pb resulted in an acceleration of the subcutaneous IOMM-Lee meningioma xenograft growth in mice.

**Conclusions:**

Overall, the current study elucidates the potential mechanism by which lead exposure promotes the proliferation of meningioma with *NF2* expression for the first time.

## 1. Introduction

With the increasing industrial application of lead, environmental contamination and human exposure to this toxic metal have become more widespread [1, 2]. Lead is known to lack physiological function and even low concentrations can be detrimental to human health [3]. Long-term exposure to lead is particularly harmful to the central nervous system (CNS) [4, 5]. It has the capacity to penetrate the blood-brain barrier and to interfere with the various functions of the CNS, thereby posing a risk for neurodevelopmental disorders during childhood development [6, 7]. Elevated blood lead concentration is considered a risk factor for tumor development within the CNS, where meningiomas are prevalent [8–10].

Meningiomas represent the most frequent primary intracranial tumors [11], accounting for approximately 30% of CNS tumors in adults and 0.4% to 4.6% in children [12, 13]. Meningiomas are classified into three grades by the World Health Organization due to their high recurrence rate and aggressiveness [14], which are associated with genetic changes and environmental factors such as lead exposure [10, 15]. A large substantial body of clinical research has demonstrated that individuals with elevated blood lead concentrations or occupational exposure to lead are at an increased risk of developing meningiomas [8, 16].

The most common genetic abnormality identified in patients with meningioma is the loss of chromosome 22.q12.2, which harbors the *neurofibromin-2* gene (*NF2*) [17, 18]. In the general population of patients with meningiomas, deletions, nonsense mutations, splice site mutations, and translocations in *NF2* are identified in 50–60% of cases [18, 19]. The gene in question encodes a protein known as Merlin, which is a 70 *k*Da protein with 10 different isoforms [17]. It function as a tumor suppressor, regulating cell processes including proliferation and intercellular contact through various signaling pathways [20]. In addition to *NF2* deficient meningiomas, a proportion of meningiomas exhibit normal *NF2* expression. The current study employs two categories of meningioma cell lines to investigate the impact of lead exposure on different types of meningiomas. The first category comprises cell lines with normal *NF2* expression (IOMM-Lee), while the second category comprises cell lines lacking *NF2* (CH157-MN). IOMM-Lee and CH157-MN are derived from patients with grade III meningiomas and with distinct genetic background [21, 22]. They are the most commonly used cell line in malignant meningiomas [23, 24].

The Hippo tumor suppressor pathway is subject to regulation at the level of its upstream components, with Merlin representing a key factor in this process. The Hippo signaling pathway plays a pivotal role in regulating cell proliferation and apoptosis, which are crucial for tumor progression [25]. In mammals, the core of the Hippo pathway comprises of a kinase cascade, mammalian sterile -like kinases 1 and 2 (MST1/2), Large Tumor Suppressor 1 and 2 (LATS1/2), Yes-associated protein (YAP) and transcriptional coactivator with PDZ-binding motif (TAZ) [26]. The transcriptional enhanced associate domain (TEAD) family of transcriptional factors serves as the primary binding partner for YAP/TAZ. In the absence of Hippo pathway signaling, YAP/TAZ undergo dephosphorylation and translocate to the nucleus where they bind to TEAD, thereby initiating transcriptional programs that promote cell proliferation, survival and migration [25, 27]. Amount studies have revealed that the loss of Merlin is associated with an increase in YAP expression and enhanced cell growth [28, 29]. Recent studies have corroborated the efficacy of ablating YAP and TAZ, or employing an inhibitor of TEAD palmitoylation, in reversing the growth of schwannomas and meningiomas [30, 31]. Further investigation is required to determine whether lead exposure-induced meningioma proliferation is mediated by the downregulation of Merlin-Hippo signaling. In the present study, we utilized two human meningioma cell lines, IOMM-Lee and CH157-MN, as *in-vitro* models, while subcutaneous injection of IOMM-Lee cells was employed to establish an *in-vivo* model. This approach aimed to elucidate the effects of lead exposure on meningioma and its underlying mechanisms.

## 2. Methods and materials

### 2.1 Chemicals and reagents

Lead trihydrate acetate (Pb(Ac)_2_, 32307) purchased from Sigma Corporation, USA. CCK8 kit (HY-K0301) from MCE Company. Cell cycle detection kit (KGA511-KGA512) was purchased from China KGI Biologics Corporation. Nuclear protein and cytoplasmic protein extraction kit (P0028) was from China Beyotime Inc. Merlin antibody (#9168), p-Merlin (Ser518) antibody (#13281), MST1 antibody (#14946), p-MST1(Thr183)/MST 2(Thr180) antibody (#49332), LATS antibody (#3477), YAP/TAZ antibody (#8418), YAP antibody (#14074), p-YAP(Ser127) antibody (#13008), and Lamin B1 antibody (#13435) purchased from CST, USA. GAPDH antibody (ab181602), sheep anti-mouse secondary antibody (ab6728) and sheep anti-rabbit secondary antibody (ab6789) were purchased from Abcam Company. Alexa Fluor-594 Phalloidin (A12381) from Invitrogen Company, USA.

### 2.2 Cell culture

IOMM-Lee cells purchased from Warner Bio-log Corporation. CH157-MN cells were gift from Dr. Xiaomin Zhang (Kunming Medical University, Kunming, China). The IOMM-Lee and CH157-MN cell lines were cultured in DEME supplemented with 10% fetal bovine serum (FBS) and 0.5% penicillin-streptomycin at 37 °C with 5% CO_2_ to maintain optimal cell growth. In accordance with the objectives of the experiment, the cells in the logarithmic growth phase were inoculated into distinct cell culture plates for utilization. The experimental group was treated with varying concentrations of lead acetate in order to ascertain the impact of lead exposure on meningioma cells.

### 2.3 Cell viability assay

The viability of the cells was determined using the Cell Counting Kit-8 (CCK-8) assay. The cells were cultured in a 96-well plate with 100 µL of DMEM containing 5,000 cells. Subsequently, the culture medium of IOMM-Lee cells added with 0, 0.5, 10, 20, 40, 80, 100, 200, 800 and 1000 µM lead acetate respectively. The CH157-MN cell culture medium was supplemented with 10, 50, 100, 200, 400, 800 and 1000 nM as well as 10 and 100 µM of lead acetate. The viability of the cells was assessed following a 24-hour exposure period. Prior to detection, 10 µL of CCK-8 solution was added to each well. The culture plate was then placed in the incubator for a period of two hours, after which the absorbance (OD) value at 450 nm was determined.

### 2.4 Cell cycle assay

A suspension of cells at a concentration of 5 × 10^5^ cells/mL was introduced into a 6-well plate, and 20 µM lead acetate was subsequently added to the medium of IOMM-Lee cell, and 200 nM of lead acetate was added to CH157-MN cell culture medium. Following a 24-hour exposure to lead, the cells were collected, washed twice with a pre-cooled PBS solution, and then fixed overnight with 70% ethanol at 4°C. After resuspension, the cells were added to propidium iodide (PI) stain and incubated at 37 °C in dark for 30 min. The cell cycle was analyzed by flow cytometry (FACSVerse, BD Biosciences, Abingdon, USA) and the results were processed using ModFit LT (Version 5.0, Verity Software House, Topsham, USA).

### 2.5 F-actin staining

After the treatment of lead, the cells were fixed with 4% paraformaldehyde for 20 minutes and subsequently washed 3 times with PBS. PBS with 0.1% Triton X-100 was employed as the membrane permeabilizing solution for 30 minutes at room temperature. Then the cells were incubated with 50 mg/mL Alexa Fluor-594-labeled phalloidin for 60 min. The remaining phalloidin was removed by washing with PBS, after which the cells were imaged using confocal microscopy (Olympus, Tokyo, Japan).

### 2.6 Western-blotting

After different treatment, the total protein was extracted using the bicinchoninic acid protein assay (Bosterbio, Pleasanton, USA). The extraction of nuclear and cytoplasmic proteins was conducted in accordance with the manufacturer’s protocol, as outlined in the kit instructions. The protein from each group was added to the SDS-PAGE gel and transferred to a PVDF membrane. TBST containing 5% dry milk was used as a blocking buffer. Then the membrane was incubated with primary antibodies at 4 °C overnight. TBST was used to clean the membrane, and the corresponding secondary antibodies were incubated for 1 h at room temperature. The target bands were quantified using a Bio-Rad ChemiDoc MP system (Bio-Rad, Hercules, USA).

### 2.7 Immunocytochemistry

The cells were rinsed with PBS and incubated with 0.3% TritonX-100 and 10% FBS at room temperature for 60 min. Then cells were exposed to the primary antibody YAP (1:200), p-H3-Ser10 (1:200) at 4 °C overnight. After the labelling of the primary antibody, the cells were washed and incubated with the second antibody at room temperature for 1 h (Alexa-488 labelled Donkey Anti-Rabbit IgG, with a dilution of 1:500). The cells were imaged using a confocal microscope (Olympus, Tokyo, Japan). For YAP cluster imaging laser light levels and detector gain and offset were set same, and all the parameters were adjusted so that no pixel values were saturated in regions analyzed. The obtained images were analyzed by Image-J 11.0 software, using a cluster-analysis plugin. Before analysis, the same input folder should specify to each image. To obtain accurate results, we conducted the YAP-cluster count in each nucleus. Therefore, we utilized DAPI to mark the position of the nucleus, framed the nucleus with ROI (region of interest), and tallied the number and area of YAP within it.

### 2.8 Xenograft mouse models

All animal experiments were conducted in accordance with the guidelines set forth by Animal Care and Welfare Committee of Jiaxing University. For the purposes of the subcutaneous xenograft studies, cells (1 × 10^7^) were inoculated into the right flank of 4–6-week-old severe combined immunodeficient (SCID) mice. All mice were randomly divided into two groups: a control group and a group treated with Pb (1000 ppm), which was administered via drinking water. The treatments were conducted for 6 weeks. Tumor volume was monitored by measuring the length (L) and width (W) of the tumor with caliper. Tumor volume was calculated using the formula (L × W^2^) × 0.5. The mice were observed twice weekly for any changes in condition and were humanely euthanized when the criteria for this were met.

### 2.9 Statistical analysis

Each experiment was conducted a minimum of three times, with the number of experiments, represented by the letter “*n*” varying depending on the specific experiment. The dose-response curves were generated using GraphPad Prism software version 7.0. The image data was analyzed using the Image-J 11.0 software. The statistical analysis of the data was conducted using SPSS 16.0 software, with the data subjected to one-way ANOVA (Dunnett T3 test). A *p*-value of less than 0.05 was considered statistically significant. The data presented in the text and figures are expressed as mean ± SEM.

## 3. Results

### 3.1 Effect of Pb treatment on meningioma cell viability

**Fig. 1:**
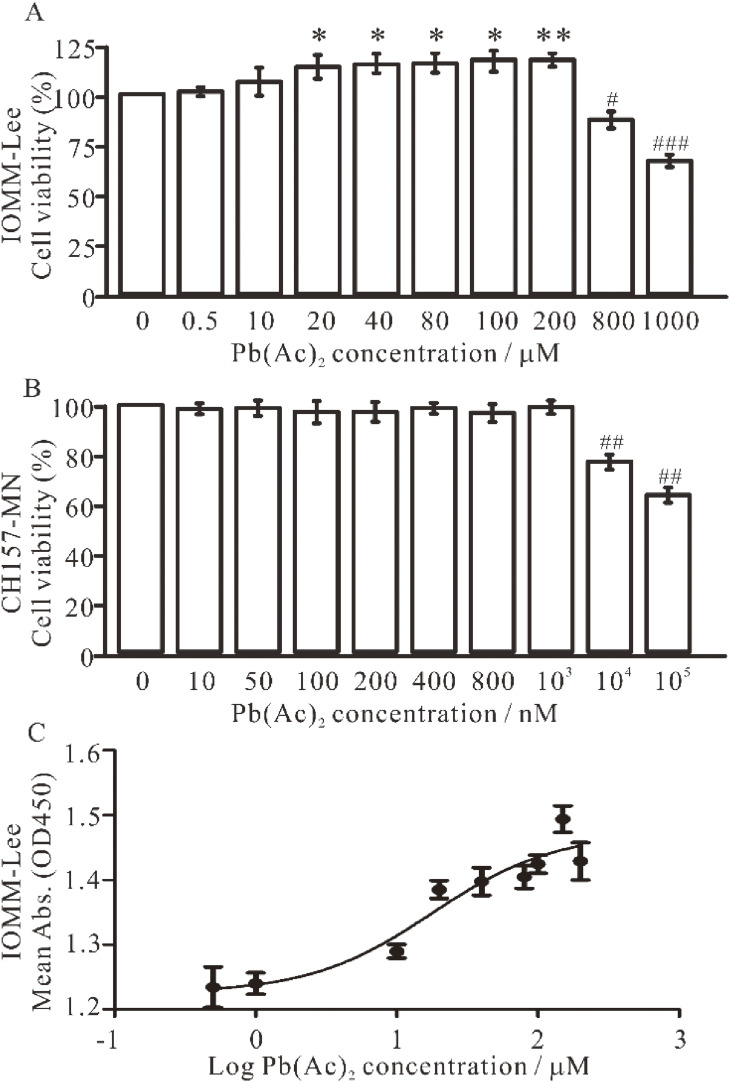
Lead acetate exposure promoted proliferation of IOMM-Lee but not CH157-MN meningioma cells. (A–B). Cells were treated with various concentrations of lead acetate for 24 h to assess cell viability. Relative percentages were calculated according to the absorbance at 450 nm in each exposure and normalized to the group without lead acetate. Data represent the mean ± SEM. Statistical differences were determined by one-way ANOVA. (C). The dose response curve of lead exposure on IOMM-Lee cells was shown, with EC_50_: indicating the concentration required for a 50% maximal effect. *n* = 3–4 (*, # *p* < 0.05; **, ## *p* < 0.01; ### *p* < 0.001).

The initial objective was to ascertain the impact of lead exposure on meningioma cell proliferation by assessing cell viability. The proliferation of the *NF2-wildtype* meningioma cell line IOMM-Lee was enhanced by lead treatment in a dose-dependent manner within a certain range (Fig. 1A). However, at concentrations exceeding 800 µM, lead acetate was observed to exert an inhibitory effect on IOMM-Lee cell proliferation. In contrary, lead exposure did not significantly affect the proliferation of the *NF2-null* meningioma cell line CH157-MN. Instead, it rendered the cell line more susceptible to growth inhibition by lead treatment. The growth of CH157-MN cells was inhibited at concentrations exceeding 10 µM (Fig. 1B). In light of the dose-dependent promotion of IOMM-Lee cell proliferation by lead exposure, we determined that the EC_50_ value for acute treatment with lead acetate was 19.6 µM (Fig. 1C). Given that a concentration of 20 µM lead acetate was observed to effectively enhance IOMM-Lee cell growth in the present study, this concentration will be employed as the primary experimental variable in subsequent investigations. Furthermore, 200 nM lead acetate was employed in the treatment of CH157-MN cells.

### 3.2 Effect of Pb treatment on meningioma cell size

**Fig. 2:**
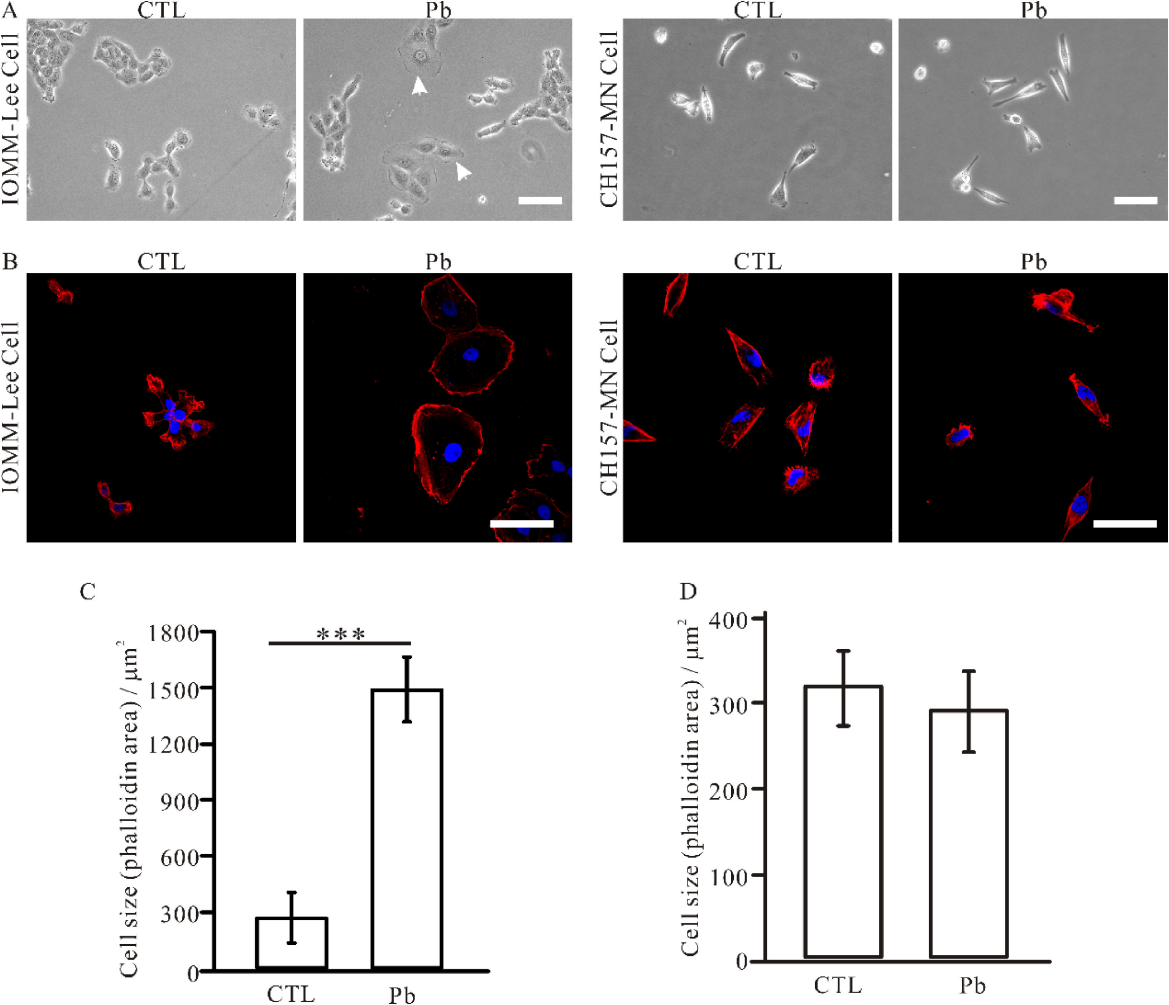
Lead exposure induced the morphological changes of IOMM-Lee cells. (A). Bright field microscopy revealed an increase in cell size upon lead treatment (white arrows), which was not observed in CH157-MN cells. (B). F-actin stained with phalloidin (red) and DAPI (blue) confirmed these findings. Scale bar, 50 µm. Quantification of the size of IOMM-Lee cells (C) and CH157-MN cells (D). Data represent the mean ± DEM. Statistical difference was determined by one-way ANOVA. *n* = 3–4 (*** *p* < 0.001).

It was observed that, in addition to promoting cellular proliferation, lead exposure also exerted an influence on cellular morphology. Following treatment with lead acetate, an increase in size was observed in IOMM-Lee cells (Fig. 2A). Conversely, no notable alterations in CH157-MN cell size were observed prior to and following lead exposure (Fig. 2A). To further confirm alternation in size observed in IOMM-Lee cells, we labelled F-actin using phalloidin to visualize its structure. The results demonstrated a substantial enlargement of IOMM-Lee cells following lead exposure, with a mean cellular size increase from 269.63 ± 67.72 µm^2^ to 1490.74 ± 87.08 µm^2^ (Fig. 2B, C). However, the CH157-MN cells exhibited minimal alterations following lead exposure (Fig. 2B, D).

### 3.3 Effect of Pb treatment on meningioma cell cycle

**Fig. 3:**
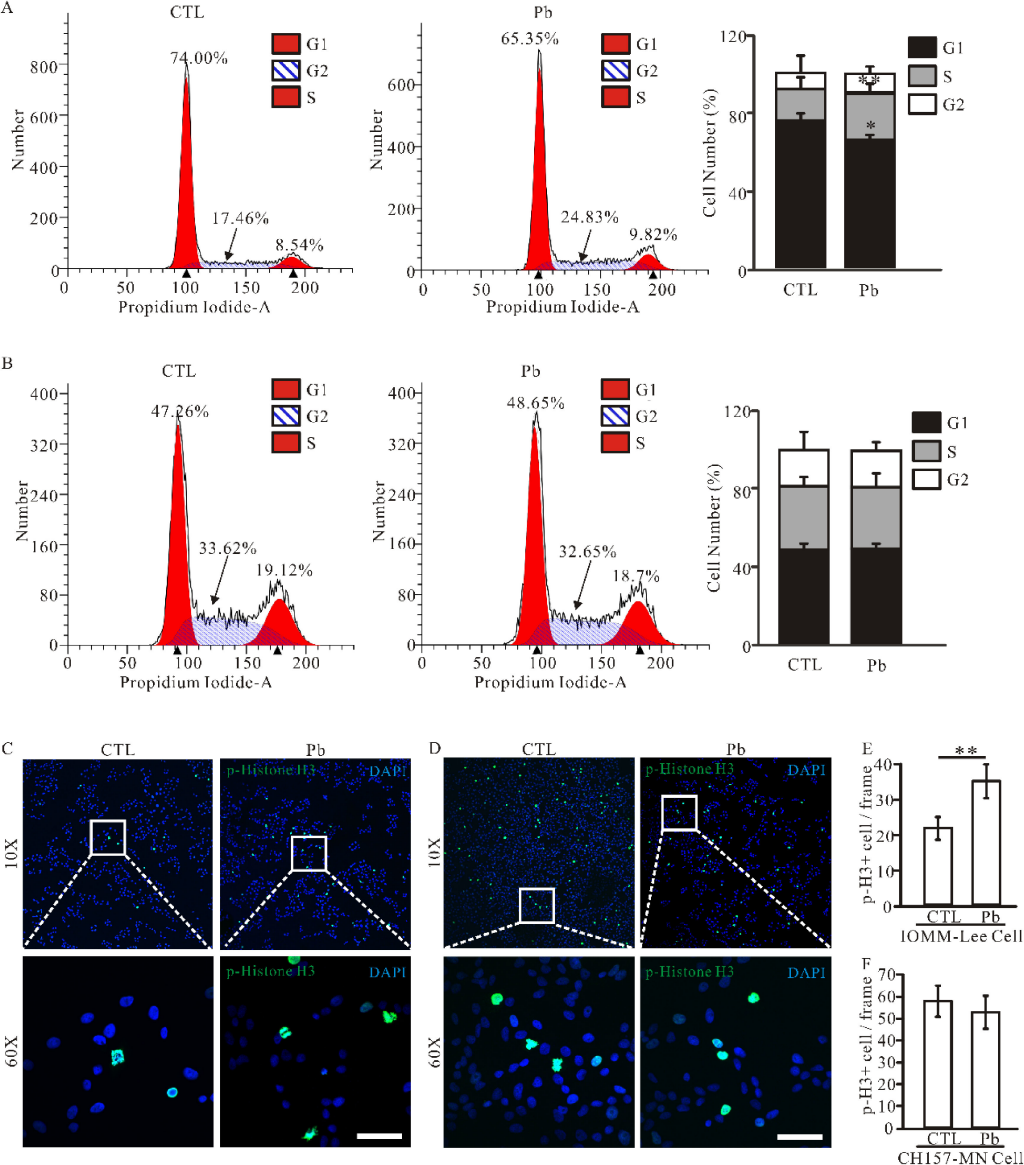
Effect of lead exposure on the cell cycle of IOMM-Lee cells. Flow cytometry was performed to analyze the cell cycle distribution in IOMM-Lee (A) and CH157-MN (B) cells. Cells treated without lead acetate were used as vehicle control, and the percentage of cells in each phase of the cell cycle is shown. Representative images show staining for p-H3 (Ser10) (Red) and cell nucleus (Blue) in IOMM-Lee cells (C) and CH157-MN cells (D). Scale bar, 50 µm. (E–F). Quantification of p-H3-positive cells was calculated from three independent experiments, and data are presented as mean ± SEM. Statistical significance was determined by one-way ANOVA test with *n* = 3–4 samples per group (* *p* < 0.05; ** *p* < 0.01).

The two meningioma cell lines were subjected to an analysis of the alterations in the cell cycle. The results showed that IOMM-Lee cells exhibited a significant alteration in the cell cycle following exposure to lead, characterized by a reduction in the G1 phase and an increase in the ration of S and G2 phases (Fig. 3A). Meanwhile, no discernible difference was observed in the CH157-MN cell cycle after lead treatment (Fig. 3B). Phosphorylated histone H3 (Ser10, pH3) is a primary marker for the mitotic phase. To further validate the alteration in cell cycle progression among meningioma cells, immunofluorescence staining was employed to assess pH3 expression following lead exposure. The results revealed an increase in pH3-positive cells per frame from 21.67 ± 3.06 to 35.00 ± 4.58 following lead treatment (Fig. 3C, E). In contrast, no change was observed in pH3 expression levels within CH157-MN cells (Fig. 3D, F). These findings collectively indicate that lead exposure exerts distinct effects on the two meningioma cell lines under investigation.

### 3.4 Pb treatment increases IOMM-Lee cell proliferation through repressing the expression of Merlin

**Fig. 4:**
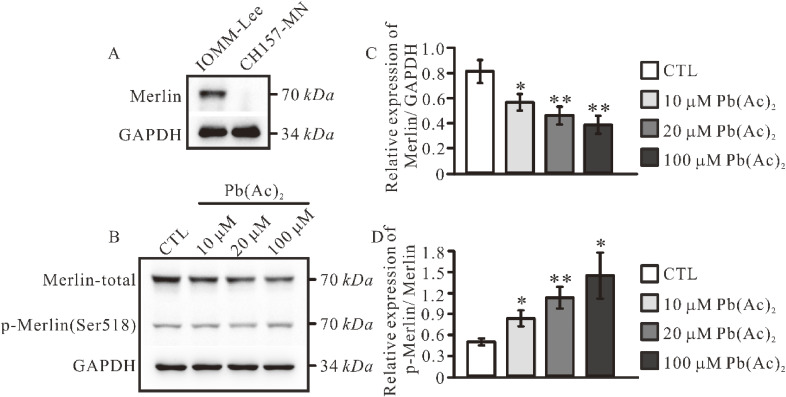
Merlin expression in meningioma cells after lead exposure. (A). Detection of Merlin protein in IOMM-Lee and CH157-MN cells. (B) Detection of Merlin and phosphorylated Merlin (Ser518) after lead treatment in IOMM-Lee cells. (C) Quantification of relative expression levels of Merlin normalized to GAPDH. (D) Quantification of relative expression levels of p-Merlin (Ser518) normalized to total Merlin. Data represents the mean ± SEM. Statistical difference was determined using one-way ANOVA test with *n* = 3–8 samples per group (* *p* < 0.05; ** *p* < 0.01).

Furthermore, studies have indicated that aberrant *NF2* gene expression represents a significant distinguishing factor between the IOMM-Lee and CH157-MN cell lines [21]. Our findings are in alignment with this conclusion (Fig. 4A). Additionally, disparities were observed between the two cell lines with respect to cellular proliferation and cell cycle dynamics (Fig. 3A, B). Prior research has demonstrated that phosphorylated merlin expression is a critical factor in the promotion of cellular proliferation [32, 33]. Western blot analysis was performed to assess merlin expression levels in IOMM-Lee cells following lead exposure. We observed that lead treatment led to a significant reduction in the expression of total merlin protein in cells, while the levels of phosphorylated merlin protein remained relatively unchanged (Fig. 4B, C). In comparison to the phosphorylated forms and total of merlin, our findings indicated a notable increase in the relative expression of phosphorylated merlin following lead exposure (Fig. 4D).

### 3.5. Pb treatment increases IOMM-Lee cell proliferation through inhibiting MST-LATS-YAP signaling pathway

**Fig. 5:**
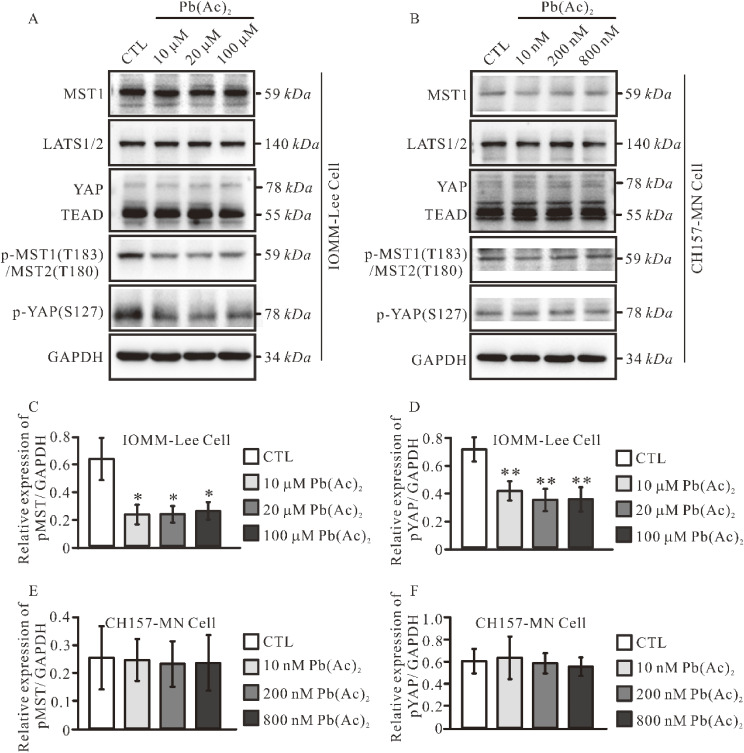
The inhibition of Hippo signaling pathway due to lead exposure in IOMM-Lee cells. (A–B). Analysis of the associated molecular expressions in IOMM-Lee and CH157-MN cells after lead treatment. Quantification of relative expression levels of p-MST1/2 and p-YAP normalized to GAPDH in IOMM-Lee cells (C–D) and CH157-MN cells (E–F). Data represent the mean ± SEM. Statistical difference was determined by one-way ANOVA. *n* = 3–4 (* *p* < 0.05; ** *p* < 0.01).

Merlin is a crucial upstream regulator of the Hippo signaling pathway [25]. Building upon previous findings, we sought to further investigate whether the expression of MST-LATS-YAP molecules in meningioma cells underwent alteration following lead treatment. In comparison to the control group, exposure to lead resulted in a notable reduction in the protein levels of p-MST1/2 and p-YAP in IOMM-Lee cells (Fig. 5A, C, D), no detectable difference was observed in CH157-MN cells (Fig. 5B, E, F).

### 3.6. YAP distribution after Pb treatment

**Fig. 6:**
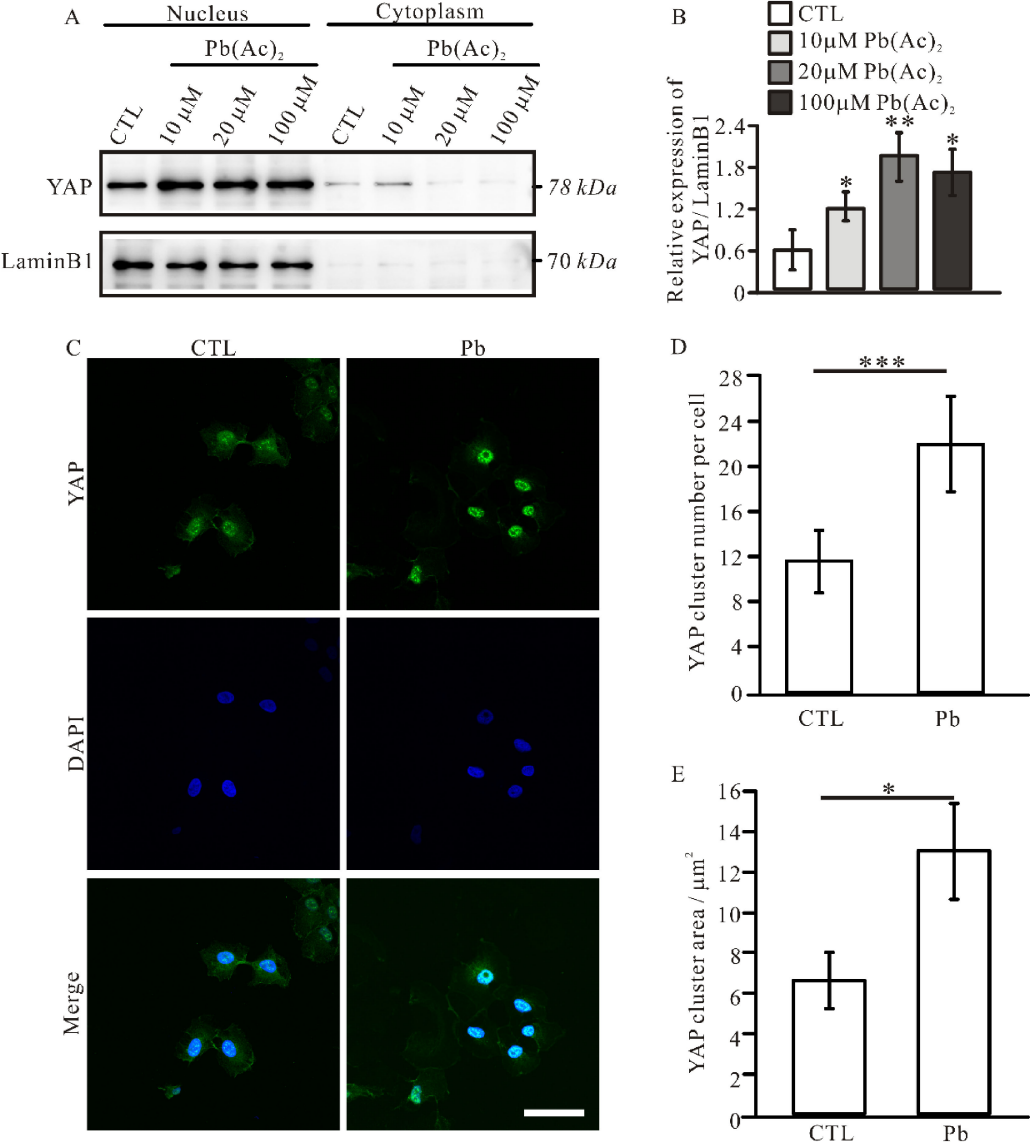
Lead exposure enhanced YAP translocation into the nucleus in IOMM-Lee cells. (A). Western blot analysis for cell nucleus and cytoplasm from different groups. (B). Quantification of relative expression levels of YAP normalized to Lamin B1. (C). Immunofluorescence staining for YAP. Representative images showing YAP cluster number in each cell (D), and the YAP cluster area (E) in control and lead treatment groups. Scale bar, 50 µm. Data represent the mean ± SEM. Statistical difference was determined by one-way ANOVA. *n* = 3–4 (* *p* < 0.05; ** *p* < 0.01; *** *p* < 0.001).

Activation of the Hippo signaling pathway led to the translocation of YAP into the nucleus, thereby facilitating cell proliferation [27]. Therefore, the assessment of YAP distribution is of paramount importance for the evaluation of Hippo pathway functionality. The nuclear and cytoplasmic components of IOMM-Lee cells from each experimental group were isolated and subsequently assessed for YAP expression levels. The results showed that a notable elevation in nuclear YAP expression following lead exposure in IOMM-Lee cells (Fig. 6A, B). To further validate these findings, immunofluorescence was employed to examine YAP distribution across all groups. Following lead treatment, there was a notable increase in the nuclear localization of YAP within IOMM-Lee cells, as indicated by an elevated number of YAP clusters per cell, from 11.78 ± 2.77 to 22.22 ± 4.29 (Fig. 6C, D). Moreover, there was a substantial increase in the area of YAP clusters from 6.61 ± 1.41 µm^2^ to 13.03 ± 2.37 µm^2^ (Fig. 6C, E). In conclusion, the presented evidence indicates that the inhibition of the Hippo signaling pathway due to lead exposure results in an increased nuclear localization of YAP within IOMM-Lee cells.

### 3.7 Effect of Pb treatment on meningioma growth *in-vivo*

**Fig. 7:**
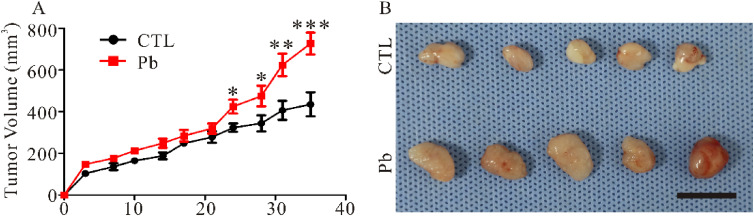
Lead exposure promotes the progression of subcutaneous meningioma xenografts in mice. (A). Subcutaneous xenograft experiments were performed with IOMM-Lee meningioma cells with or without Pb. (B). Tumor volume of subcutaneous xenografts on day 30 after the treatment of Pb. Scale bar, 1 cm. Data represent the mean ± SEM. Statistical difference was determined by one-way ANOVA. *n* = 8–10 (* *p* < 0.05; ** *p* < 0.01; *** *p* < 0.001).

To summarize the functional role of Pb during meningioma progression, we subcutaneously injected IOMM-Lee meningioma cells to construct the subcutaneous xenograft mouse model. The growth of the IOMM-Lee meningioma xenograft was monitored weekly basis by measuring the volume of the tumor. The administration of Pb resulted in an acceleration of the subcutaneous meningioma xenograft growth in mice (Fig. 7A). The mean tumor volume of the control group was 435.05 ± 57.71 mm^3^, while that of the Pb group was 727.16 ± 52.69 mm^3^ at day 30, indicating a significant increase (Fig. 7B).

## 4. Discussion

A large number of clinical studies have revealed an association between lead exposure and an elevated risk of meningioma development [10]. However, the precise underlying mechanism remains elusive. The present study aimed to investigate the effect of lead exposure on two human meningioma cell lines, namely IOMM-Lee and CH157-MN. The results demonstrated that lead treatment facilitated the proliferation of IOMM-Lee cells, whereas no notable effect was observed on CH157-MN cells. We also demonstrated a statistically significant increase in IOMM-Lee meningioma mouse flank tumor size with 1000 ppm Pb treatment. The Merlin-Hippo signaling pathway may serve as a pivotal regulator of the growth of *NF2-wildtype* meningiomas induced by lead exposure.

The most prevalent endogenous factor contributing to the development of meningiomas is the *NF2* gene mutation [18]. Nevertheless, approximately half of meningioma patients express *NF2*, indicating that meningioma occurrence is the result of multiple etiological factors. In examining the influence of environmental elements on meningioma formation, it is imperative to ascertain the role of Merlin. The study conducted by Yu Mei examined single nucleotide variants and somatic copy number variations in human meningioma cell lines. It was observed that the loss of chromosome 22 occurred in CH157-MN but not in IOMM-Lee. Furthermore, the findings suggest that IOMM-Lee meningioma cells may not originate directly from mutations in the *NF2* gene [21]. Consequently, a significant amount of research has commonly utilized the IOMM-Lee and CH157-MN cell lines to investigate the effects of drugs and compounds on tumor proliferation due to their specific *NF2* expression. This approach is more clinically relevant than manual inhibition of *NF2* expression in cells [34]. The current study demonstrated that lead primarily regulates meningioma cell proliferation through Merlin. Consequently, the two cell lines with different *NF2* expressions exhibited disparate results. This underscores the fact that meningioma growth is the result of a combination of factors and demonstrates how individuals with diverse genetic backgrounds respond differently to similar environmental exposures.

Our results demonstrated that the expression of Merlin in IOMM-Lee cells was significantly inhibited following lead treatment. The primary function of Merlin is to link the transmembrane protein to the actin cytoskeletons, which is involved in cell volume regulation, proliferation and migration [35]. Properly folded Merlin protein suppresses tumor growth. Clinical studies have reported that nonsense or frameshift mutations of *NF2* result in truncated protein products that generally have more severe phenotypes [17, 36]. The study from Waldt, N et al. revealed that after the knockdown of *NF2* gene in IOMM-Lee cells, there was an increase in cell size, loss of contact inhibition, and further increase proliferation [34]; However, overexpression of this gene reduced meningioma cell growth [37]. Consequently, we propose that lead treatment leads to a reduction in Merlin expression, which simultaneously contributes to increased cell size and promotes cellular proliferation. However, due to the absence of Merlin expression, CH157-MN exhibits no significant impact on either cell size or proliferation.

In our previous studies, we found that lead exposure of IOMM-Lee cells promoted cell proliferation through the Merlin-mTOR signaling pathway, where Merlin expression decreased and mTOR was activated [38]. Overexpress of Merlin could effectively correct the proliferative effects of lead acetate on IOMM-Lee cells. In this study, we used the IOMM-Lee and CH157-MN two meningioma cell lines, which have different *NF2* gene expression. We found that lead exposure specifically promotes *NF2-wildtype* meningioma cell proliferation and increases cell size, but has no effect on *NF2-null* meningioma cells, further confirming that a critical role for Merlin in mediating the effects induced by lead treatment (Fig. 1, 2, 7).

In addition, phosphorylation of histone H3 molecules is an important marker for cells entering the mitotic phase [39]. We observed a significant increase in the expression of phosphorylated histone H3 expression following lead treatment (Fig. 3C, E). This finding, combined with previous observations of increased cell volume and G2/S phase in the cell cycle of meningioma cells after lead exposure, suggests that lead may facilitate an increase in meningioma cells entering the mitotic phase, ultimately leading to significant cell proliferation. Previous studies have shown that lead acetate acts as a mitogen for mouse renal proximal tubule cells and can stimulate endothelial cell proliferation [40, 41]. Given that meningioma cells are primarily derived from subarachnoid endothelial cells, these results suggest that lead may have similar effects on meningioma cells.

Merlin regulates cellular activities through various downstream signaling pathways such as the Hippo signaling pathway, which is widely implicated in tumor proliferation [27, 42]. Liyam Laraba et al. reported that genetic ablation of the Hippo effectors YAP and TAZ and application of TEAD palmitoylation inhibitors suppressed the Hippo signaling pathway and regressed schwannoma and meningioma tumor growth both *in-vivo* and *in-vitro* [30]. In our results, we observed that lead exposure inhibited the expression of p-MST and p-YAP in *NF2-wildtype* cells (Fig. 5) but showed no change in *NF2-null* CH157-MN cells (Fig. 5), suggesting that lead treatment alters the Hippo pathway through Merlin molecular specifically within meningiomas.

Furthermore, Merlin negatively regulates YAP as an upstream activator of the Hippo signaling pathway; thus, deficiency of Merlin leads to the activation of YAP, resulting in increased nuclear translocation of YAP, where it associates with TEAD transcription factors in the nucleus [43, 44]. We exclusively observed Hippo pathway alternation and YAP nuclear translocation in IOMM-Lee but not CH157-MN cells (Fig. 5), suggesting that the Merlin-Hippo axis primarily regulates the effects induced by lead exposure. Clinical studies revealed that *YAP* fusion, particularly with *MAML*2, occurs in a subset of pediatric *NF2-wildtype* meningiomas, and the *YAP1-MAML2* fusion is similar to *NF2* mutant meningioma [45–47]. However, with the discovery and analysis of more *YAP* fusion meningioma cases, certain subtypes such as *YAP1-FAM118B* fusion, these meningiomas exhibit distinct biological characteristics from *NF2* mutant meningioma [48]. In conclusion, the YAP molecule is a significant effector molecule in the development of meningioma. The Merlin molecule can promote the development of meningioma by regulating the YAP molecule, but Merlin might not be the sole factor influencing YAP regulation based on different fusion genes of *YAP*.

In our previous investigations, we observed that IOMM-Lee cells exhibited activation of the mTOR signaling pathway following lead treatment [38]. Numerous studies have established a significant interaction between the Hippo and mTOR signaling pathways [49, 50]. Upon DNA damage, the Hippo pathway activates MST1, which not only inhibits Akt signaling but also interacts with RHEB (Ras homologue enriched in brain) to suppress mTORC1 signal transduction, thereby promoting apoptosis [51]. Concurrently, components of the Hippo pathway, specifically LATS1/2, can directly engage with mTORC1 to inhibit its activity [52]. When cell density reaches excessive levels, cells activate LATS1/2 to downregulate mTORC1 and ultimately curtail proliferation [52]. The YAP/TEAD inhibitors such as verteporfin can effectively inhibit YAP function and subsequently attenuate the mTOR signaling pathway [53, 54]. In this study, we found that lead exposure resulted in inhibition of the Hippo signaling pathway in IOMM-Lee cells, evidenced by decreased phosphorylated MST1 levels and enhanced nuclear YAP signaling (Fig. 4, 5). These alterations may concurrently influence the mTOR pathway leading to its activation and subsequent promotion of cellular proliferation.

Although some studies have reported that lead ions can bind to metallothionein *in vivo* and be transported into the cell via divalent metal transporters [55, 56], there is currently no evidence for a direct interaction between lead ions and Merlin. Therefore, it is more plausible that lead exposure acts on Merlin through indirect intermolecular mechanisms. Previous research has shown that lead exposure promotes DNA synthesis and accelerates the cell cycle in astrocytoma cells by activating protein kinase C (PKC) and mitogen-activated protein kinases [57, 58]. Transforming growth factor-β inhibited membrane associated protein (TIMAP) is abundant in endothelial cells and its phosphorylation is regulated by PKC [59, 60]. Merlin is a potential substrate of TIMAP which further regulates its expression and distribution. Taken together, these studies suggest that while lead exerts a proliferative effect on different types of cells, the underlying molecular mechanisms may vary.

## 5. Conclusion

Our results suggested that lead exposure promoted *NF2-wildtype* but not *NF2-null* meningioma cells proliferation. In particular, our data showed that lead inhibited the Merlin-Hippo signaling pathway and activated YAP translocated into the nucleus then promoted cell growth in IOMM-Lee cells. These results contributed to the understanding of the association between lead exposure and meningioma.

## Declarations

### Availability of data and material

The datasets analyzed during the current study are available from the corresponding author on reasonable request.
